# Fundamental changes in the antimicrobial resistance profile of *Klebsiella quasipneumoniae* ATCC 700603 in response to meropenem stress

**DOI:** 10.1186/s12866-025-04100-6

**Published:** 2025-06-26

**Authors:** Mai H. Elmahdy, Ahmed F. Azmy, Tarek Dishisha, Ahmed O. El-Gendy, Mohamed Sebak

**Affiliations:** Department of Pharmaceutical Microbiology and Immunology, Faculty of Pharmacy, Beni-Suef University, Beni-Suef, 62514 Egypt

**Keywords:** *Klebsiella quasipneumoniae*, Antimicrobial resistance, Meropenem, Carbapenems, Evolutionary engineering, Stress factor

## Abstract

**Background:**

*Klebsiella* is one of the most challenging superbugs having a high tendency to acquire rapid resistance to many antibiotics, even the ones recognized as the last resort. In several hospitals and environmental niches, *Klebsiella* is continuously exposed to residual amounts of antibiotics at sub-inhibitory concentrations forming an environmental stress motivating them to adapt and evolve antimicrobial resistance. In the present study, meropenem (MEM) resistance was induced experimentally in a MEM-sensitive strain of *K. quasipneumoniae* ATCC 700603 through sequential sub-culturing in presence of sub-inhibitory concentrations of MEM over a period of 20 days. To uncover the possible mechanisms standing behind the evolution of antimicrobial resistance upon successive exposure to stress of MEM rather than horizontal gene transfer (HGT) of antibiotic resistance genes.

**Results:**

Fully adapted cells of the 20th generation (G20) showed MEM-resistance with elevated minimum inhibitory concentration (MIC) by 256-fold compared to the parent cells (G0). The main mechanism of resistance was the production of carbapenemases, which was assured by different tests including nitrocefin, modified-Hodge test (MHT), and modified carbapenem inactivation method (mCIM). The degradation of MEM reached 65.93% by the produced carbapenemases of G20 as determined by the HPLC analysis. Transcriptomics analysis of the class D carbapenemase encoding gene, *bla*_*OXA-2*_, revealed that it was significantly over-expressed by a 3.12-fold (*p* < 0.05) in G20 compared to G0.

**Conclusion:**

The evolved MEM resistance aroused mainly from MEM degradation by carbapenemases, neither increased efflux nor decreased influx of MEM. The rational use of antibiotics is essential to reduce bacterial exposure to the environmental basal levels of antibiotics and decreasing the evolution of antimicrobial resistance.

**Graphical Abstract:**

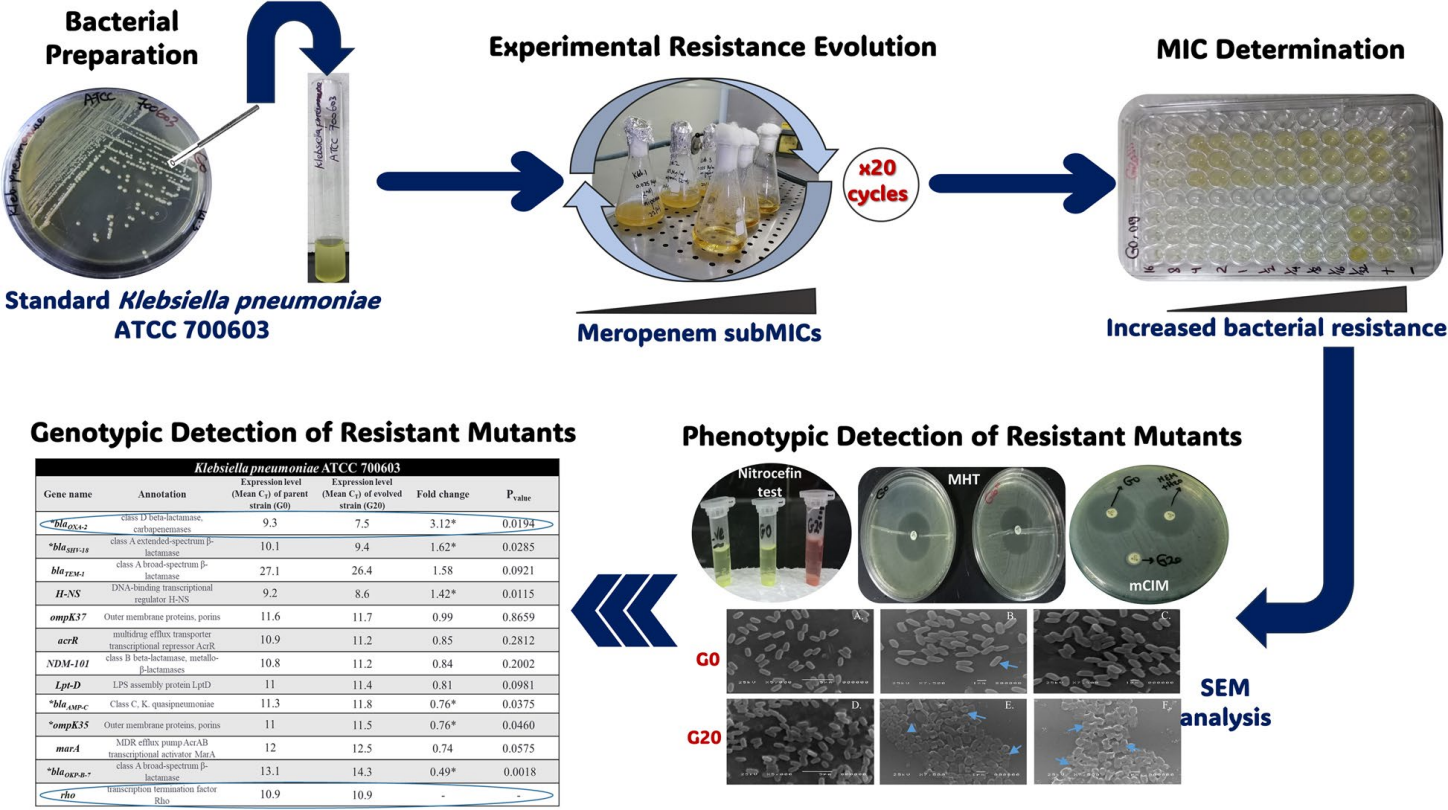

**Supplementary Information:**

The online version contains supplementary material available at 10.1186/s12866-025-04100-6.

## Introduction

There is a significant disparity between the development of new antibiotics and the spread of antimicrobial resistance (AMR), favoring AMR [1]. Nearly, 700,000 deaths are recorded globally every year due to infections with multidrug-resistant (MDR) bacteria [2]. By 2050, 10 million fatalities are expected globally by AMR infections, according to the UK government's Antibiotic Resistance Study [3] Regrettably, the development and dissemination of carbapenem resistance (CR) is a matter of global concern with critical emergencies, as carbapenems are the last-resort antibiotics [4]. Recently, carbapenem-resistant *K. pneumoniae* (CRKP) have occupied the second order among the most prevalent top ten MDR organisms (MDROs) at the intensive care units [5, 6].

The mis- and overuse of antibiotics makes all living creatures at risk even animals and livestock owing to rendering the environment always containing basal levels of antibiotics which are presented to nature via three major pathways; human/animal excretions (urine and feces), pharmaceutical and agricultural industry wastes, and improper discharge of unused or expired antibiotics [7, 8]. Continuous exposure of bacteria to these environmental low-level antibiotic doses with sub-inhibitory concentrations constitutes stress on the bacterial cells stimulating them to mutate and acquire AMR for survival [7]. This follows the theory known as the mutant selection window [9–11]. Furthermore, these residual levels of antibiotics have led to further spread of different types of MDROs due to the cross-resistance phenomenon [12].

Many informative reports manifested the effect of exposure to sub-minimum inhibitory concentration (sub-MIC) of antibiotics on the bacterial resistance pattern, mutation rates, gene transcription, and protein expression. However, most of them used limited exposure time, usually 24–48 h, on already clinically resistant strains harboring antibiotic-resistant genes (ARGs) [7, 13, 14]. The current study scrutinizes the incremental induction of MEM resistance in the MEM-sensitive strain, *K. pneumoniae* ATCC 700603 through 20-day exposure to sub-MICs of MEM.

In the present study, *Klebsiella quasipneumoniae* subsp. *similipneumoniae* (ATCC 700603), formerly known as *Klebsiella pneumoniae* K6 that was isolated from the urine of a hospitalized patient in 1994 in the USA, was used as the reference/parent strain [15]. This microorganism’s genome was fully sequenced by Elliott and his colleagues, containing a chromosome and two plasmids, *pKQPS1* and *pKQPS2* [15], and is a CLSI quality control strain for antimicrobial susceptibility testing. Moreover, it is a recommended quality control strain for antimicrobial susceptibility testing of β-lactamases’ production [16, 17]. The strain is also sensitive to MEM which is an essential issue for detection the effect of MEM stress on the AMR pattern of bacteria in our study. The effect of acquired MEM resistance on other antibiotic classes was also investigated. Furthermore, our study clarified the possible mechanisms of emerging MEM resistance in sensitive microorganisms which reflects what the natural stress faced by bacteria in the environment.

## Materials and methods

### Bacterial strain and growth conditions

*K*. *quasipneumoniae* ATCC 700603 was used as the parent strain. The antibiogram of this strain was verified by applying the Kirby-Bauer disk diffusion method as recommended by the Clinical and Laboratory Standards Institute (CLSI) [18] (Supplementary Figure S1).

### Experimental evolution of antibiotic resistance by successive exposure

Firstly, the bacterial cells of the wild-type parent strain were refreshed from frozen glycerol stock into nutrient broth (NB) with a ratio of 1% (v/v) and incubated overnight at 37 °C. Fifty microliters of grown cells were then streaked out on nutrient agar and incubated at 37 °C for another 24 h. One pure colony was cultivated into 50 ml NB in a 250 ml flask and incubated at 37 °C for 20 h. Consequently, 500 µl of bacterial suspension were aseptically sub-cultured into 50 ml fresh NB medium containing MEM with a final concentration of 0.05 µg/ml (0.5 MIC) in a 250 ml flask. Cells were serially sub-cultured with the same manner in presence of stepwise increasing concentrations of MEM (0.05, 0.1, 0.2, 0.3, 0.4, 0.5, 1, 2, 4 and 8 µg/ml, respectively) in a liquid medium over a period of 20 days [19]. Notably, each generation were exposed to the respective MEM concentration twice. Subsequently, cells from each generation were streaked on nutrient agar supplemented with the proper concentration of MEM for isolation of pure colonies. Negative control cultures of *K. quasipneumoniae* 700,603 were grown in parallel in the absence of MEM. All cultures either test or controls were conducted in triplicate. Also, each sub-culture, either broth or colonies, was conserved in glycerol stocks for later analysis.

### Assessment of induced antimicrobial resistance

#### Determination of MEM MIC

The MIC of MEM was measured for each generation to verify that the evolution of the AMR pattern was successfully achieved. MIC values were determined using the CLSI guidelines for the broth micro-dilution method [18]. Briefly, the wells of a 96-well plate were filled with 100 µl phosphate-buffered saline (PBS) (pH 7.4), except for the first well in which 200 µl of 32 µg/ml MEM solution was added. Then, two-fold serial dilutions of MEM were performed using PBS achieving MEM concentrations ranging from 0.0625–32 µg/ml. Bacterial cultures of different generations (G0-G20) equivalent to 0.5 McFarland were subsequently diluted 100-fold with the cation-adjusted Mueller Hinton broth (CAMHB), prepared as double-strength. Finally, 100 µl of these bacterial cultures were seeded into serial MEM concentrations.

Wells of positive growth control containing 100 µl PBS and 100 µl bacterial suspension were prepared to confirm normal bacterial growth. Also, wells containing PBS and plain double-strength CAMHB were used as negative controls to test the sterilization conditions. Microtiter plates were incubated at 37 °C for 18 h and inspected visually to determine MICs [20]. The lowest antibiotic concentrations at which no discernible bacterial growth can be detected by visual inspection was recorded as the MIC value [21]. All MIC assays were run in triplicates. The net MIC was expressed as the mean of 3 MIC values of MEM for each generation.

#### Detection of MIC of other antibiotics

Cells of the parent- (G0) and evolved strains (G20), were examined for their antimicrobial susceptibility against antibiotics from different classes for investigating the cross-resistance phenomenon. The susceptibility was determined by measuring the MIC of each antibiotic utilizing the broth microdilution method. Then, the interpretation for sensitivity was managed using breakpoints as reported in CLSI for each antibiotic in case of *K. pneumoniae*. The antibiotics were used with the following concentration range: ampicillin/sulbactam (8/4—128/64 µg/ml), cefazoline (8–256 µg/ml), cefoxitin (2–512 µg/ml), cefotaxime (0.5–64 µg/ml), cefepime (0.5–256 µg/ml), gentamicin (4–128 µg/ml), amikacin (4–128 µg/ml), ciprofloxacin (0.5–64 µg/ml), levofloxacin (0.5–64 µg/ml), in addition to MEM (0.125–32 µg/ml). The assays were performed in three independent replicates.

#### Effect of MEM on growth of wild-type and adapted K. quasipneumoniae

The growth curves of the different *K. quasipneumoniae* generations were plotted and examined to evaluate their viability upon exposure to sub-MICs of MEM and ensure the adaptation [22]. Glycerol stocks of (G0, G5, G10, G15, and G20) were refreshed by overnight cultivation at 37 °C in tryptone soya broth (TSB) containing no MEM in the case of unstressed cells (G0) or supplemented with sub-MICs concentrations of MEM (0.2, 0.4, 2, and 8 µg/ml, respectively) for the respective generations [7]. Fifty microliters from each cultivation were transferred to 5 ml of fresh Luria Bertani (LB) broth, which was then incubated at 37 °C for 3 h until the mid-log phase. Importantly, each tested generation was diluted 100 folds with LB medium in absence of MEM, and presence of MEM with low concentration (0.4 µg/ml) and high concentration (8 µg/ml) to obtain a full image of bacterial growth and viability normally and under antibiotic stress. Finally, 200 µl of each generation were aseptically transferred to a sterile 96-well microtiter plate and incubated overnight at 37 °C [23].

The optical density (OD_620_) was measured every 2 h over the cultivation period after shaking in the microtiter plate reader. Bacterial cells of *K. quasipneumoniae* grown without MEM were considered as positive growth controls. Growth curves were generated by plotting OD_620_ values versus time (h). All experiments were run in independent triplicates.

The different growth phases were identified using a graph depicting the relationship between Ln OD and cultivation time (t). The slope of the curve during the logarithmic phase represents the maximum specific growth rate (*μ*_*max*_) for each generation.

- Maximum specific growth rate (*μ*_*max*_) (1/h) = *(Ln OD*_*2*_*– Ln OD*_*1*_*)/(t*_*2*_*– t*_*1*_*)*
- Duplication time (*t*_*d*_) (h) = *Ln (2)/μ*_*max*_

### Phenotypic detection of resistance mechanisms and adaptation methods

#### Detection of β-lactamases’/carbapenemases’ activities

##### Nitrocefin hydrolysis test

Half McFarland solutions were freshly prepared from the overnight culture of the parent strain (G0) and the stressed (G20). Equal volumes of the bacterial suspension and the nitrocefin solution (0.5 mM) were mixed and the immediate color change was followed. A change within 1–2 min to a maximum of 5 min indicates strong β-lactamase activity, while a longer duration ˃ 10 min indicates weak activity [24]. A mixture of PBS with nitrocefin solution was used as a negative control.

##### Modified Hodge test (MHT)

The MHT was carried out for the cells of G0 and G20 as explained elsewhere [25]. In brief, the MEM sensitive indicator strain, *Escherichia coli* ATCC 25922, was prepared as 1/10 turbidity of half McFarland. Ten micrograms MEM disc was aseptically located in the middle of the plate. Finally, 2–3 pure colonies of G0 and G20 were individually streaked starting from the disc proximity towards the Petri-dish periphery in a straight line. After 24-h incubation at 37 °C, the plates with cloverleaf-like growth of *E. coli* were concluded as positive carbapenemase producers [26].

##### Modified carbapenem inactivation method (mCIM)

Initially, a loopful of an overnight culture of G0 and G20 cells was transferred to a 400 µl distilled water. Afterwards, 10 µg-MEM disc was aseptically impregnated in the bacterial suspension of each test strain. All tubes with MEM discs were incubated at 37 °C for 2 h as a carbapenem inactivation step. Negative control tubes were prepared from MEM disc immersed in water with no bacterial cells. MEM discs were carefully removed and transferred to the surface of a Mueller Hinton agar (MHA) plate containing a lawn of half McFarland *E. coli* ATCC 25922. The plates were incubated at 37 °C for 18–24 h. Measurements were made for the diameters of the resulting zones of inhibition [27, 28].

#### Detection of MEM degradation via HPLC analysis

MEM level was analyzed in the blank culture medium and the medium inoculated with G20 cells using the reversed phase-high performance liquid chromatography (RP-HPLC). For this, evolved G20 colonies were inoculated into LB media containing sub-MIC of MEM (8 µg/ml) and incubated overnight at 37 °C. Aliquots of the resulting culture were then centrifuged twice at 10,000 xg for 15 min to produce cell-free supernatants (CFS). The standard samples of MEM (8 µg/ml), MEM/LB, and CFS were analyzed by HPLC after being thoroughly filtered through a 0.45 µm nylon syringe filter. All samples were analyzed in triplicate. The analysis started by injecting 100 µl of each sample into the HPLC instrument (Agilent 1260 Infinity II LC, Agilent Technologies, USA). Separation of the compounds was performed using a reversed-phase chromatographic column (Agilent Zorbax C-18, 250 mm × 4.6 mm × 5 µm). The mobile phase contained 30 mM monobasic phosphate buffered water (pH 3)/acetonitrile mixture at a fixed ratio of 80:20 and flowing rate of 1 ml/min for isocratic elution with a total run time of 10 min. MEM was detected by an UV (λ = 298 nm), and a diode array detector (DAD) [29].

#### Detection of efflux activity by ethidium bromide-agar cartwheel method

Tryptone soya agar (TSA) plates containing ethidium bromide (EtBr) (0, 0.5, 1, 1.5, 2, and 2.5 µg/ml, respectively) were freshly prepared, divided into six sectors by radial lines forming a cartwheel pattern, and secured from light. Overnight cultures of G0, G5, G10, G15, and G20 cells were prepared for evaluation of their efflux activity.

Bacterial cell suspension of each generation was adjusted to approximately 10^6^ cells/ml and swabbed onto the EtBr-agar plates in one direction starting from the central circle of the wheel towards the periphery of the plate. Each plate included a minimum of one negative efflux activity strain as a fluorescent comparative control. Plates were finally wrapped with aluminum foil and incubated at 37 °C for 18 h [30]. Subsequently, EtBr-TSA plates were inspected for fluorescence under UV light using a UV transilluminator. The minimum concentration of EtBr which reveals fluorescent bacterial growth was recorded for each generation.

#### Bacterial visualization using scanning electron microscopy (SEM)

Both unstressed cells (G0) and fully stressed ones (G20) were visualized using scanning electron microscope for observing any morphological alterations of the cell before and after exposure to the MEM stress factor. Overnight broth cultures of G0 and G20 were freshly prepared in absence and presence of 8 µg/ml MEM, respectively. The bacterial cells were harvested via centrifugation at 5,000 xg for 10 min. The pellets were washed three times with 100 mM PBS (pH 7.4), air dried, and fixed by immersing the slides in 2.5% (v/v) phosphate-buffered glutaraldehyde for 1 h at 4 °C. Cells were washed again three times with PBS, dehydrated by passing through incremental concentrations of ethanol–water mixtures (10, 30, 50, 70, 90, and 100%) for 5 min each. After being subjected to critical point drying, the specimens were firmly attached to the aluminum specimen stubs using double-sided sticky tapes. Finally, samples were subjected to gold–palladium coating with a sputter coater, and then visualized under a GM-5200 electron microscope (JOEL, Tokyo, Japan) with a voltage of 25 kV and a magnification power ranging from × 5,000 to × 7,500 [31].

#### Detection of biofilm formation

The biomass within the biofilm was measured using the previously published crystal violet (CV) staining method [32, 33] with slight modifications. Briefly, overnight cultures of G0, G5, G10, G15, and G20 were grown at 37 °C. Cells were then diluted 1/100 in TSB containing 1% glucose and sub-MIC of MEM for each generation. A negative control containing only TSB was used. Then, 200 µl from each generation was added to the sterile 96-well flat-bottomed microtiter plates which were incubated at 37 °C for 48 h. After aspiration of the media, cells were washed three times with 100 mM PBS (pH 7.4) to remove non-adherent cells. The formed biofilm was fixed by heating at 60 °C for 1 h. The biomass within the biofilm was stained with 1% CV for 10 min. Excess CV was eliminated by thoroughly washing the plate five times with distilled water, which was then dried at 60 °C for 10 min. Finally, 200 µl of 33% glacial acetic acid was added to each well for 30 min to resolubilize adsorbed CV, and the absorbance was measured at OD_570_ using microtiter plate reader.

### Genotypic detection of resistance mechanisms

#### Determination of antibiotic resistance genes (ARGs)

The ARGs were determined using different databases reported in the following review article [34]. Relying on being last reviewed and updated, both the Comprehensive Antibiotic Resistance Database (CARD) (https://card.mcmaster.ca/) [35] and the ResFinder database (CGE Server (dtu.dk)) [36] were chosen. Especially, the Resistance Gene Identifier (RGI) tool inside the CARD was used for determination of the ARGs of *K. quasipneumoniae*. In addition, 5 metallo-β-lactamases genes including *IMP*, *VIM*, *NDM*, *OXA-48*, and *KPC* were also examined in the genome of *K. quasipneumoniae.*

#### Primer design

Each ARG's primer pair was created using Primer3. *ver. 4.1.0* [37]. The design was based on the reported nucleotide coding sequence in the GenBank database (www.ncbi.nlm.nih.gov/nucleotide). The primers were ordered from (Willowfort, Birmingham, England). Primers used for ARGs, and metallo-β-lactamases genes are presented in Supplementary Tables S3 and S4.

#### RNA extraction and quantitative real time-PCR (qRT-PCR)

The total RNA of the parent strain (G0) and the evolved strain (G20) were extracted from the cells at mid-log phase of growth [38]. Pellets were harvested and resuspended in RNA lysis buffer for RNA extraction following the manufacturer’s protocol enclosed with ABT Total Mini Extraction Kit (Applied Biotechnology, Egypt). Pure RNA extract was stored at −20 °C for further analysis after 24 h by qRT-PCR.

The expression levels of the ARGs of G0 and G20 were quantitatively measured by qRT-PCR using one step *HERA* SYBR® Green RT-qPCR Kit (Willowfort, Birmingham, England). The amplification reaction of each gene was carried out with a total volume of 20 µl as follows; 10 µl of 2X *HERA* SYBR® Green RT-qPCR master mix, 1 µl of 20X RT enzyme mix, 1 µl of each forward and reverse primers, 3 µl of RNA template, and 4 µl of nuclease-free water. The amplification program started with reverse transcription for cDNA synthesis at 53 °C for 15 min, and enzyme inactivation at 95 ^°^C for 5 min, followed by 40 cycles of denaturation at 95 °C for 10 s and annealing/extension at 60 °C for 30 s using Real Time PCR (DTlite, DNA-Technology, Russia). The expression levels of the investigated ARGs were normalized by using the constitutively expressed *rho* (transcription termination factor Rho) gene as an internal housekeeping gene [39]. 5ˋ-AACTACGACAAGCCGGAAAA-3ˊ and 3ˊ-ACCGTTACCACGCTCCATAC-5ˋ were used as forward and reverse primers for *rho* control gene, respectively with a product size of 99 bp. The level of each gene’s expression was measured in triplicate and compared to the steady-state expression of the *rho* gene, and the mean of cycle threshold (Ct) values were determined. Using the 2^−ΔΔCt^ method, fold changes and relative expression levels of the ARGs were calculated [40].

### Statistical analysis

All experiments were done in three independent replicates and the presented data are the mean values ± standard deviation (SD). A one-way analysis of variance (ANOVA) was first conducted to assess the significance of the mean values of the dataset, followed by a Student's t-test to determine significant differences between individual treatment means, with a p-value threshold of < 0.05, unless otherwise specified [32].

## Results

### Effect of progressive exposure to antibiotic on MIC

**Fig. 1:**
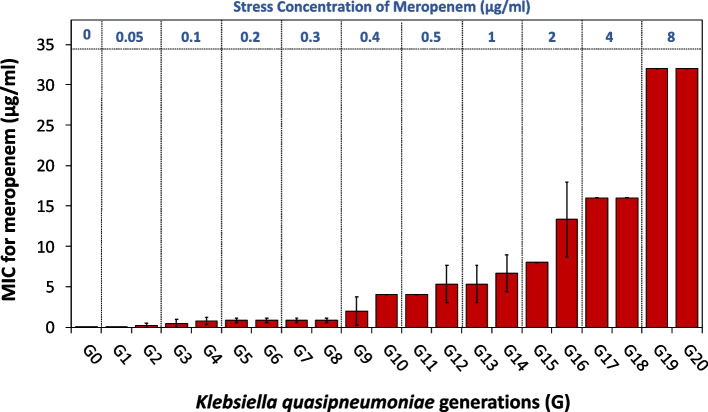
Effect of meropenem (MEM) stress on MIC values along different generations (G) of *K*. *quasipneumoniae* ATCC 700603. The applied MEM stress concentration in μg/ml is shown on top of the figure. The presented data are the mean of three independent replicates ± SD. The increase in MIC values along different generations was statistically significant at *p* < 0.05

MEM’s MIC value in the unstressed cells (G0) was 0.125 µg/ml, while the MIC values in the initial 5 generations (G1-G5) were increased by eightfold from 0.125 µg/ml to 1 µg/ml. The MIC values of the next 5 generations (G6-G10) were raised by 32-fold compared to the parent strain reaching 4 µg/ml at which *K. quasipneumoniae* became resistant to MEM according to the CLSI guidelines. Additionally, the MIC values of the third 5 generations (G11-G15) were increased by 64-fold reaching 8 µg/ml. Interestingly, after 20 generations, the MIC of the stressed G20 reached 32 µg/ml which was 256-fold compared to the unstressed G0 (Fig. 1). Values of SubMIC of MEM used for resistance evolution and the resulted MIC were recorded in Supplementary Table S1.

### Evaluation of cross-resistance with other antibiotics

The antimicrobial susceptibility of the parent strain and evolved strain was evaluated against a panel of 9 antibiotics from different classes. The tested antibiotics involved 5 β-lactam antibiotics other than MEM. Non β-lactam antibiotics included gentamicin, and amikacin as old and new aminoglycosides, respectively, along with ciprofloxacin and levofloxacin as representative of 2nd and 3rd generations of quinolones, respectively.

The MEM-stressed cells showed cross-resistance towards all β-lactam antibiotics evaluated which was indicated by elevation of the AMR level through the increase of their MIC values by 8- to 16-fold compared to the unstressed ones (Table 1). It is worth mentioning that in some cases, the AMR pattern was totally changed. According to the CLSI guidelines, the stressed cells converted from either sensitive or intermediate to resistant in the case of cefepime and ampicillin/sulbactam, respectively. In contrast to non β-lactam antibiotics in which the stressed cells showed no changes in MIC values compared to the unstressed ones (Table 1).

**Table 1:** Investigation of cross-resistance pattern of *K*. *quasipneumoniae* ATCC 700603 to other β-lactam and non β-lactam antibiotics as a result of MEM stress

**Antibiotic**	CLSI breakpoints of MIC (µg/ml) *			**Mean of MIC value**		**Fold increase in MIC value**	_**Changes in antimicrobial susceptibility**_
	**S**	**I**	**R**	**(G0)**	**(G20)**		
**Ampicillin/Sulbactam**	≤ 8/4	16/8	≥ 32/16	16	128	8	I➔➔R
**Cefazolin**	≤ 2	4	≥ 8	128	>256	>2	R➔➔R
**Cefoxitin**	≤ 8	16	≥ 32	32	>256	8	R➔➔R
**Cefotaxime**	≤ 1	>2	≥ 4	4	32	8	R➔➔R
**Cefepime**	≤ 2	-	≥ 4	1	16	16	S➔➔R
**Gentamicin**	≤ 2	4	≥ 8	16	16	No change	R➔➔R
**Amikacin**	≤ 4	8	≥ 16	2	1	No change	S➔➔S
**Ciprofloxacin**	≤ 0.25	0.5	≥ 1	0.25	0.25	No change	S➔➔S
**Levofloxacin**	≤ 0.5	1	≥ 2	1	1	No change	I➔➔I
**MEM**	≤ 1	2	≥ 4	0.125	32	256	S➔➔R

^*^ S = Sensitive, I = Intermediate, R = Resistant

### Evaluation of bacterial growth and viability under MEM stress

**Fig. 2:**
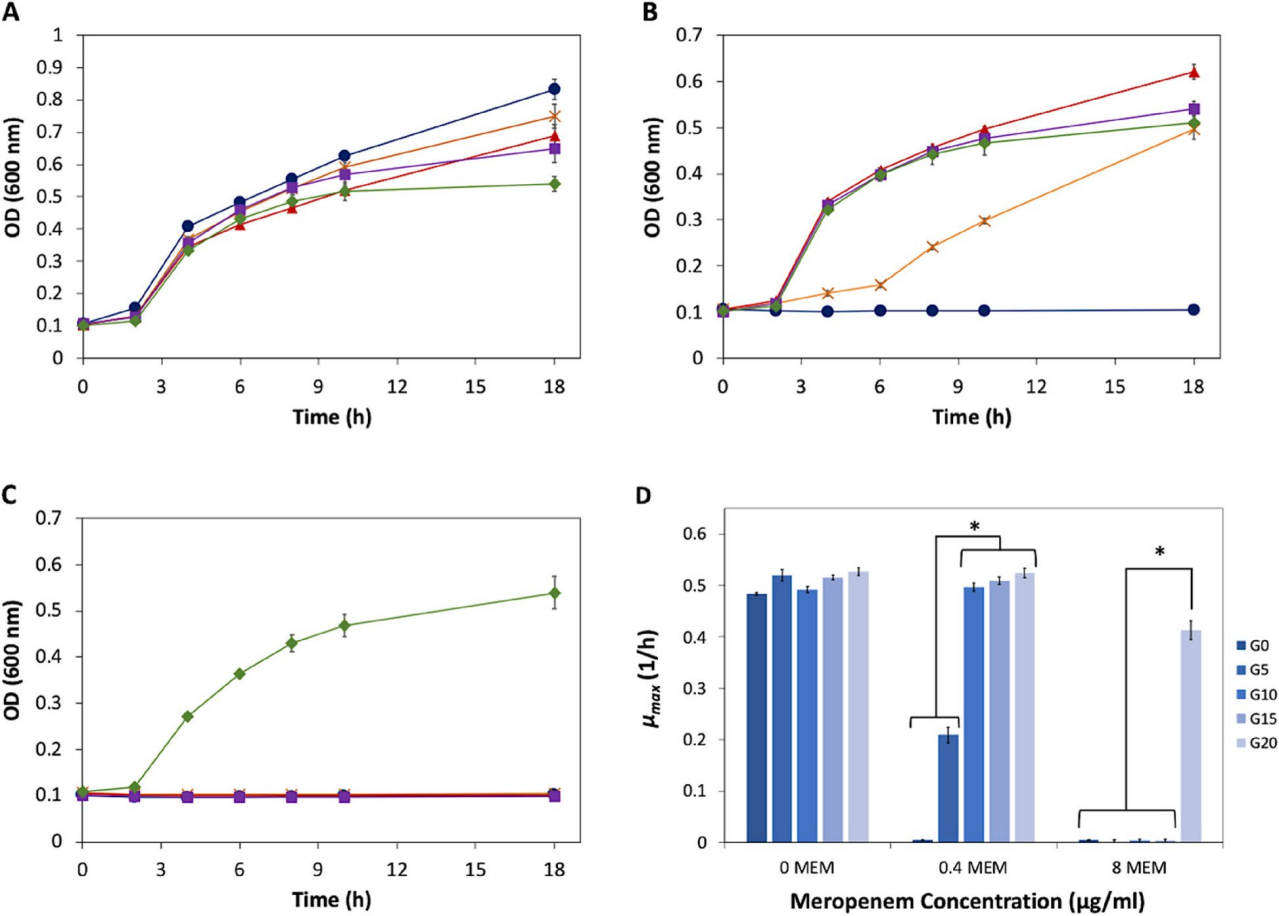
Effect of meropenem (MEM) stress on the growth of wild-type and adapted generations (G) of *K*. *quasipneumoniae* ATCC 700603, showing the growth of different generations in presence of (**A**) 0 µg/ml of MEM, (**B**) 0.4 µg/ml of MEM, and (**C**) 8 µg/ml of MEM; (●) for G0, (×) for G5, (▲) for G10, (■) for G15, and (◆) for G20. **D** Comparison of the maximum specific growth rates (*µ*_*max*_) for different generations of *K*. *quasipneumoniae* ATCC 700603 exposed to different concentrations of MEM stress. (*) indicates statistically significant difference at *p* < 0.05

Growth curves for all generations were monitored in the absence of MEM (0 µg/ml) and in the presence of 0.4 and 8 µg/ml MEM (Fig. 2A–C). Two key parameters were evaluated: final cell density and maximum specific growth rate (µmax). In the absence of MEM, all generations displayed an initial lag phase lasting approximately 2 h, followed by a logarithmic growth phase between 2 and 4 h, and subsequently a deceleration and stationary phase (Supplementary Figures S2 and S3). The *µ*_*max*_ values were comparable across generations, averaging 0.51 ± 0.02 h⁻^1^ (duplication time, *t*_*d*_ ~ 1.37 h) (Fig. 2D). The final optical density at 600 nm was highest in generation G0 (0.832), gradually decreasing in successive generations, reaching a minimum of 0.540 in G20 (Fig. 2A). The most notable difference was observed during the deceleration and stationary phases, where growth of the adapted G20 cells was reduced by approximately 36.5% compared to the parental G0 strain.

Under low MEM stress (0.4 µg/ml), only generations G10, G15, and G20 maintained a normal growth pattern similar to that observed without MEM, with *µ*_*max*_ values around 0.51 ± 0.01 h⁻^1^ (*t*_*d*_ ~ 1.36 h) (Fig. 2D). In contrast, G0 cells were completely unable to grow (*µ*_*max*_ = 0). G5 cells exhibited an initial slow growth phase (*µ* = 0.06 h⁻^1^, *t*_*d*_ = 11.55 h) lasting for 6 h, followed by a period of faster growth (*µ* = 0.21 h⁻^1^, *t*_*d*_ = 3.30 h) from 6 to 8 h, and eventually transitioned to a deceleration phase between 8 and 18 h (*µ* = 0.08 h⁻^1^, *t*_*d*_ = 8.66 h) (Fig. 2D and Supplementary Figure S3). Final OD values were similar for G15 and G20 (OD = 0.526), with G10 showing a slightly higher OD of 0.621.

When the MEM concentration was increased to 8 µg/ml, only G20 cells were capable of growth, with a *µ*_*max*_ of 0.41 h⁻^1^ (*t*_*d*_ = 1.69 h) and a final cell density of 0.539, nearly identical to values observed under both 0 and 0.4 µg/ml MEM conditions (Fig. 2A-C). This strongly indicates that G20 cells had adapted to MEM stress, demonstrating successful antimicrobial resistance (AMR) evolution.

Growth curves for each generation under the different MEM concentrations (0, 0.4, and 8 µg/ml) are shown individually in Supplementary Figure S2. Corresponding *µ*_*max*_ values are reported in Supplementary Table S2.

### Phenotypic detection of resistance mechanisms

#### Production of β-lactamases/carbapenemases

**Fig. 3:**
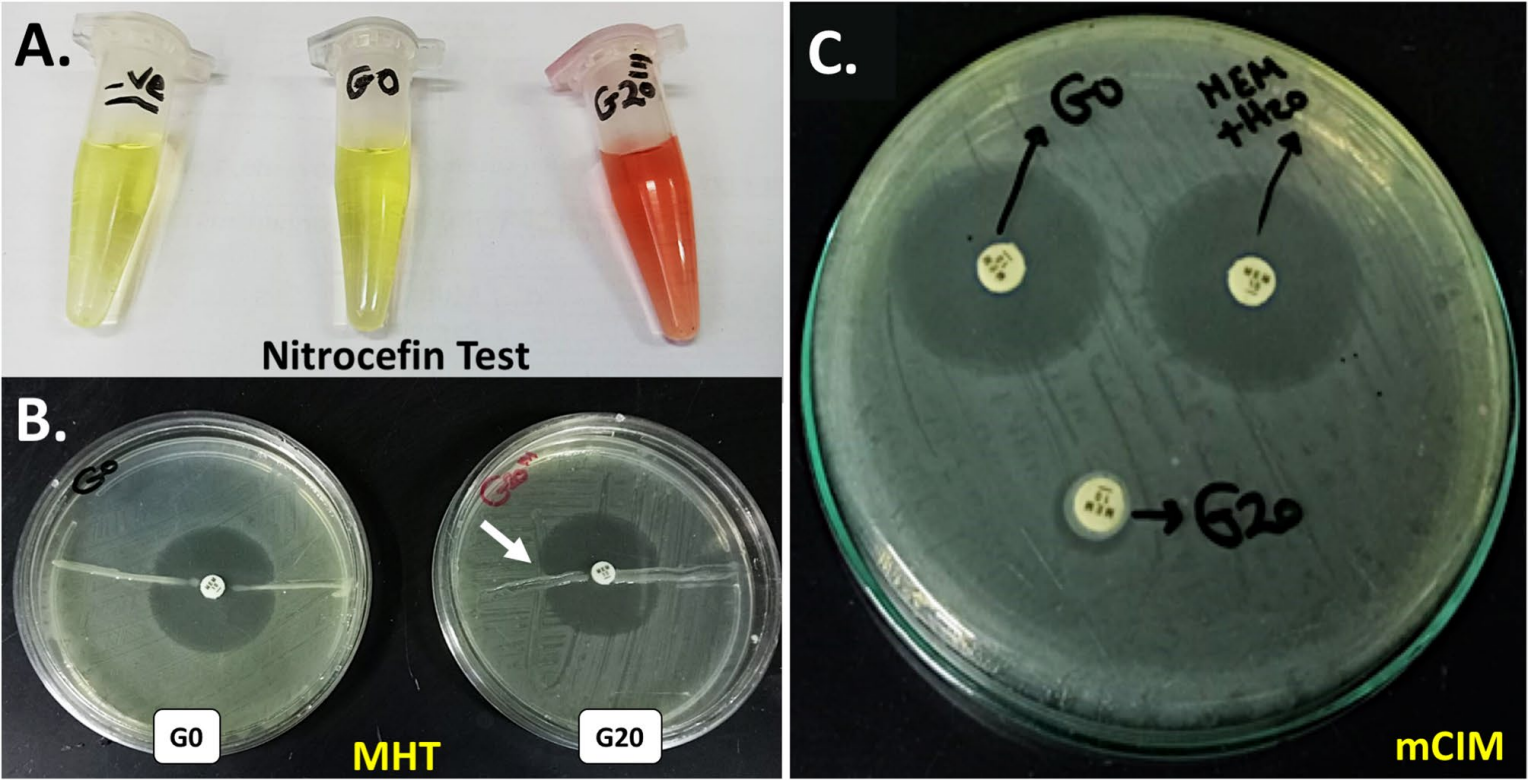
Phenotypic detection of carbapenemases production from *K*. *quasipneumoniae* ATCC 700603 due to MEM stress through three tests; (**A**) nitrocefin, (**B**) Modified Hodge test (MHT) in which the white arrow indicates the characteristic cloverleaf-like indentation of *E. coli* growth due to positive carbapenemase production from the stressed cells of G20, and (**C**) Modified carbapenem inactivation method (mCIM)

Both unstressed cells (G0) and stressed cells (G20) were screened for the production of β-lactamases through three different phenotypic detection methods; nitrocefin, MHT, and mCIM. Initially, the yellow color of nitrocefin started to change to red upon its addition to the fully stressed cells of G20 reaching its maximum intensity within 5–7 min and remained stable for hours later indicating its strong β-lactamase activity. On the other hand, parent G0 cells showed no color change (Fig. 3A).

MHT is the most extensively used phenotypic method for the investigation of carbapenemases [41]. The evolved G20 cells exhibited a characteristic cloverleaf-like indentation of the *E. coli* growth along its streaking confirming a positive carbapenemase production. To the contrary, the G0 cells showed a normal zone of inhibition without notable indentation along the streaking of the G0 cells (Fig. 3B).

mCIM is considered the most recent phenotypic screening method of carbapenemases compared to nitrocefin test and MHT. MEM discs previously incubated in bacterial suspension of G0 showed clear inhibition zones of the *E. coli* growth with 34 mm diameter (Fig. 3C). Therefore, the unstressed cells of G0 were elucidated as negative carbapenemase producers, while discs previously incubated in G20 bacterial suspension showed very small halo zone of approximately 7 mm diameter indicating MEM inactivation with carbapenemase produced from the stressed G20 cells [27].

#### Detection of MEM degradation via HPLC

**Fig. 4:**
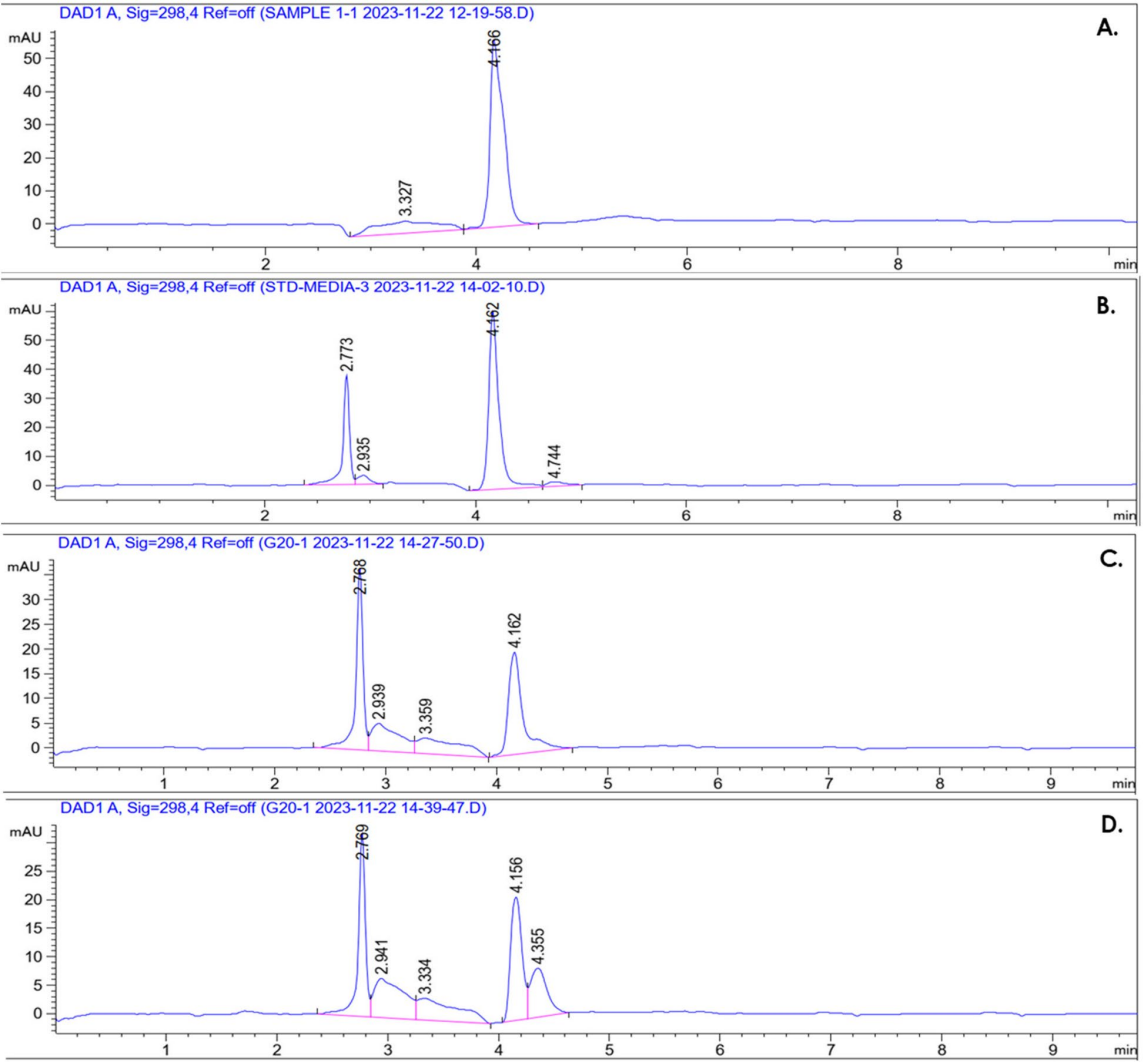
Investigation of MEM degradation by the fully stressed G20 cells through RP-HPLC technique. **A** Chromatogram of standard MEM (8 µg/ml). **B** Chromatogram of MEM/LB media. **C**, **D** Chromatogram of test samples of CFS of evolved G20 cells in which MEM was eluted at 4.162, and 4.156 min

Samples involving standard MEM, MEM/LB, and cell free supernatant (CFS) were respectively injected and analyzed by the RP-HPLC technique. Standard MEM with a concentration of 8 µg/ml was first analyzed and eluted at 4.166 min (Fig. 4A). Subsequently, a negative control sample composed of plain media with MEM was analyzed a MEM elution peak was observed at 4.162 min (Fig. 4B). Finally, the test samples containing MEM/media additionally with well-filtered CFS of fresh G20 cells were analyzed. MEM of the test samples was eluted with a retention time of 4.162, and 4.156 min. The area under the curve (AUC) of the MEM peak in the test samples was highly reduced by approximately 65.93% when compared to its counterpart in the negative control (Fig. 4C, D).

#### Detection of efflux activity

**Fig. 5:**
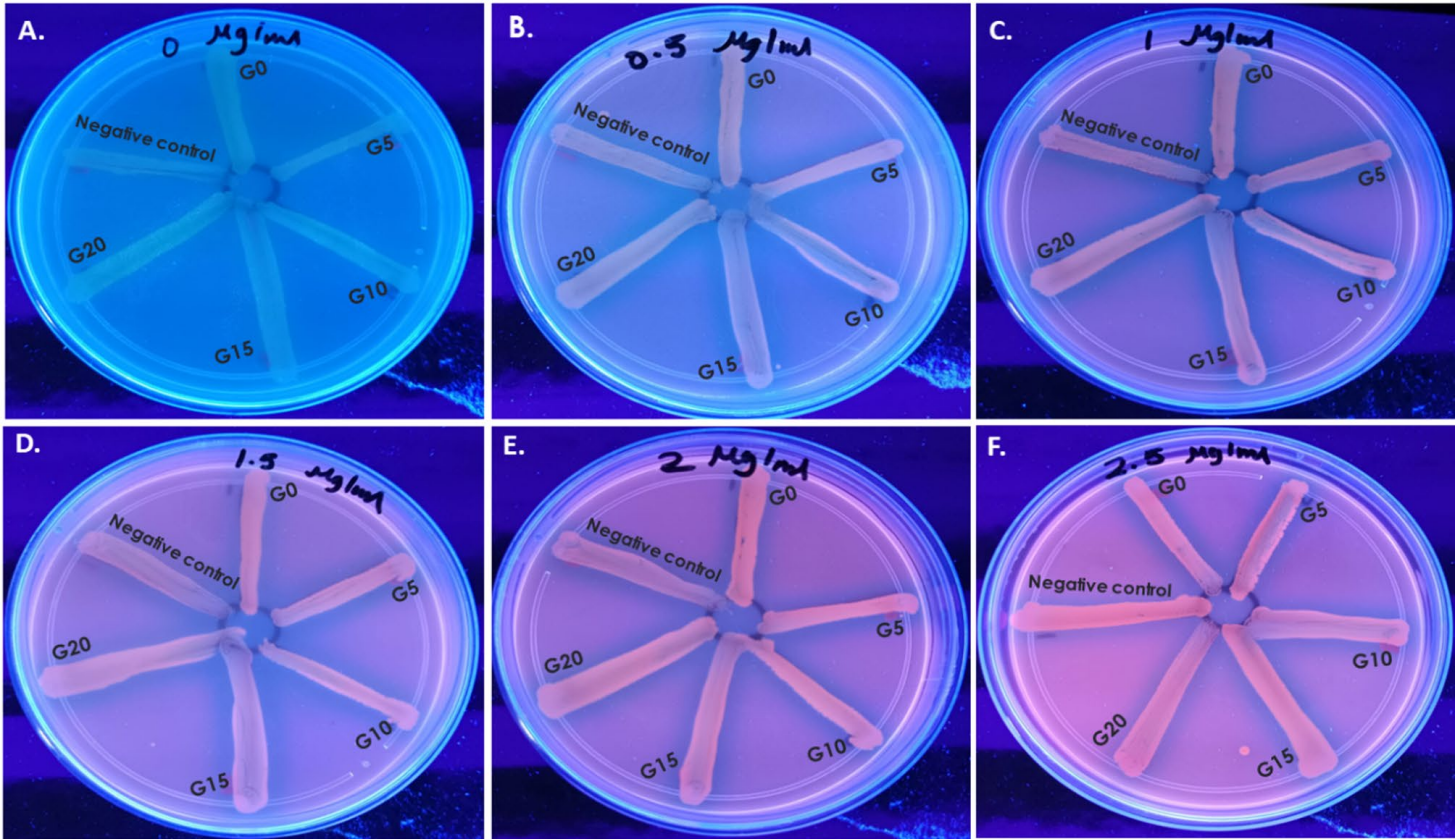
Phenotypic detection of efflux activity by EtBr-agar cartwheel method in which different generations of *K. quasipneumoniae* ATCC 700603 (G0, G5, G10, G15, and G20) in addition to negative control were exposed to increasing concentrations of EtBr, 0—2.5 µg/ml. **A** 0 µg/ml EtBr, **B** 0.5 µg/ml EtBr, **C** 1 µg/ml EtBr, **D** 1.5 µg/ml EtBr, **E** 2 µg/ml EtBr, and **F** 2.5 µg/ml EtBr

All tested generations of *K. quasipneumoniae* were fluorescent even at the minimum EtBr concentration, 0.5 µg/ml, as the comparative negative control. This result meant that all generations did not possess any efflux activity (Fig. 5).

#### Effect of MEM stress on cell morphology and biofilm formation

**Fig. 6:**
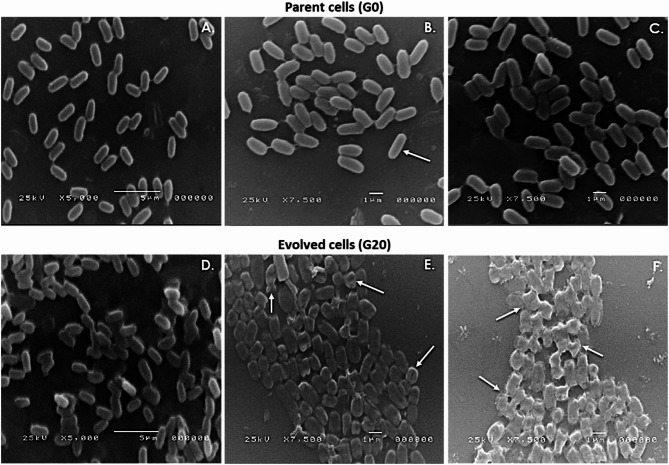
Scanning electron micrographs of parent G0 cells and evolved G20 cells. **A**, **B**, and **C** belonged to the parent cells of G0. Arrow in B indicates the typical morphology of *K. quasipneumoniae* exhibiting the rod-shaped cells with smooth surfaces. **D**, **E**, and **F** belonged to the evolved cells of G20. Arrows in E and F indicate irregularities in cell surface and possible disintegrations of the evolved cells with increased tendency to aggregate into clusters. Evolved cells appeared more rounded and swelled with rough surfaces containing holes and grooves. Cell wall disintegrations, deformities, and lysis are more prominent in F. Both magnification power; X5,000 and X7,500, were used for better visualization

SEM analysis of MEM-stressed G20 cells showed distinguished morphological alterations compared to the unstressed cells (G0). G0 cells exhibited the original morphology of *K. quasipneumoniae* as typical rod-shaped cells with smooth surfaces (Fig. 6A-C). To the contrary, the evolved cells of G20 appeared are more rounded, swelled, rough surface as variable irregularities on cell surfaces, as well as cell wall disintegration. Bacterial surface irregularities ranged from individual bumps, cavities, and grooves to overall deformed cells. Cell wall disintegration was detected as corrugation, holes, or even cell wall lysis. Additionally, some lysed and fused cells were gathered to each other as clusters (Fig. 6D-F).

**Fig. 7:**
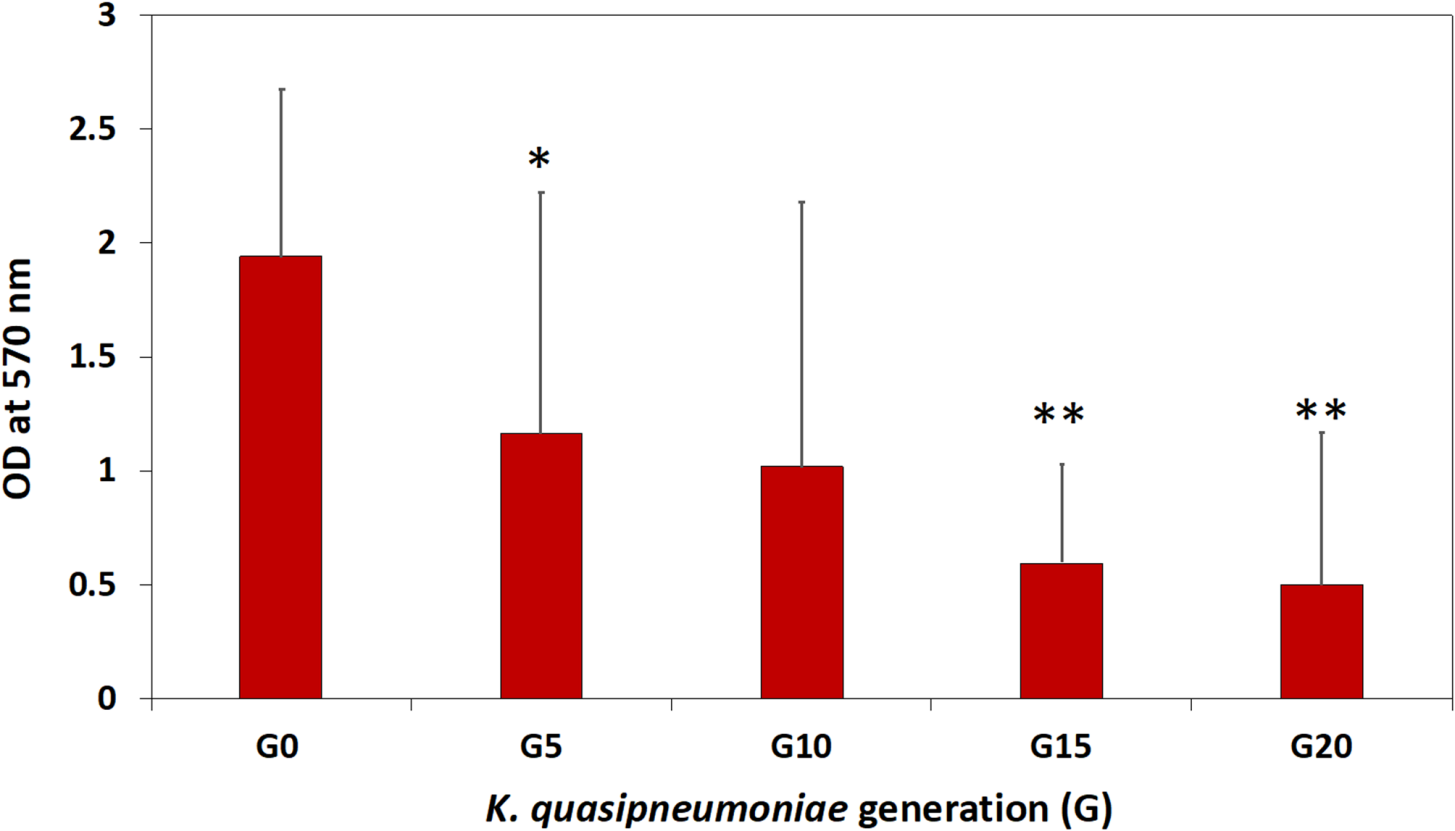
Effect of MEM stress on biofilm-forming capabilities of *K*. *quasipneumoniae* ATCC 700603 as determined from using the crystal violet assay. In case of G5, the decrease in biofilm formation was significant at *p* < 0.1 compared to that at G0, which was indicated by (*). In higher generations of G15, and G20, the decrease in biofilm formation was significant at *p* < 0.01 compared to that at G0, which was indicated by (**)

Additionally, MEM stress affected the biofilm-forming capabilities of *K. quasipneumoniae*. MEM inhibited the ability of evolved generations (G5-20) to form biofilm compared to the unstressed cells of G0 (Fig. 7). Thus, an inverse correlation between sub-MIC values of MEM and biofilm formation capabilities of different generations was found.

### Genotypic detection of resistance mechanism using qRT-PCR

Among the 12 ARGs detected, some were upregulated, while others were downregulated under the MEM stress. Hence, determination of the expression level of each differentially expressed gene of the ARGS before and after MEM adaptation was a necessity. Notably, repeated exposure of *K. quasipneumoniae* to sub-inhibitory concentrations of MEM resulted in a significant upregulation in the expression of *bla*_*OXA-2*_ by a 3.12-fold increase (*p* < 0.05) in G20 cells compared to G0 cells. To some extent, *bla*_*SHV-18*_ and *H-NS* genes were moderately upregulated by 1.62- and 1.42-fold increase, respectively. Conversely, MEM stress caused a significant downregulation of *ompK35* and *bla*_*AMP-C*_ with a 0.76-fold decrease in the expression of both genes. Additionally, the expression of *bla*_*OKP-B-7*_ was moderately downregulated by a 0.49-fold decrease. However, the change in the expression of the other differentially expressed genes, was not statistically significant (*p* ≥ 0.05) (Table 2).

**Table 2:** Quantitative RT-PCR for detection of relative expression levels and fold changes of ARGs in parent cells (G0) and evolved cells (G20)

Gene	Class	Expression level of G0 ^a^	Expression level of G20 ^a^	Fold change	Regulation	*P*_value_
*bla*_*OXA-2*_	Class D beta-lactamase, carbapenemases	9.3	7.5	3.12*	up	0.0194
*bla*_*SHV-18*_	Class A extended-spectrum β-lactamase	10.1	9.4	1.62*	up	0.0285
*bla*_*TEM-1*_	Class A broad-spectrum β-lactamase	27.1	26.4	1.58	up	0.0921
*H-NS*	DNA-binding transcriptional regulator H-NS	9.2	8.6	1.42*	up	0.0115
*ompK37*	Outer membrane proteins, porins	11.6	11.7	0.99	down	0.8659
*acrR*	Multidrug efflux transporter transcriptional repressor AcrR	10.9	11.2	0.85	down	0.2812
*NDM-101*	Class B beta-lactamase, metallo-β-lactamases	10.8	11.2	0.84	down	0.2002
*Lpt-D*	LPS assembly protein LptD	11	11.4	0.81	down	0.0981
*bla*_*AMP-C*_	Class C, *K. quasipneumoniae*	11.3	11.8	0.76*	down	0.0375
*ompK35*	Outer membrane proteins, porins	11	11.5	0.76*	down	0.0460
*marA*	MDR efflux pump AcrAB transcriptional activator MarA	12	12.5	0.74	down	0.0575
*bla*_*OKP-B-7*_	Class A broad-spectrum β-lactamase	13.1	14.3	0.49*	down	0.0018
*rho*	Transcription termination factor Rho	10.9	10.9	-	-	-

The asterisk (*) indicates statistically significant difference at *p* < 0.05

^a^ Mean C_t_

## Discussion

The evolution of the AMR is one of the greatest public health concerns that appeared since the dawn of the antibiotic era [19]. Although huge efforts were exerted to decrease the AMR, the rate of its prevalence rises day by day [21]. Hence, the gap between the rapid emergence of AMR and the slow detection of new antibiotics or even modification of them has been highly expanded [1]. Importantly, the most six challenging MDR nosocomial human pathogens are gathered in the term “ESKAPE superbugs” which refer to *Enterococcus faecium*, *Staphylococcus aureus*, *K*. *pneumoniae*, *Acinetobacter baumannii*, *Pseudomonas aeruginosa*, and *Enterobacter* spp. [21].

*K. pneumoniae,* not only is one of the terrible ESKAPE superbugs, but it is also classified in the list of critical priority pathogens, certainly among the highest three bacteria with global concern according to the WHO in 2017 [1, 5, 42, 43]. The cause refers to the high tendency of *K. pneumoniae* to evolve rapid resistance to many antibiotics even to the last resort ones such as colistin and MEM, known as carbapenem-resistant *K. pneumoniae* (CRKP) [1, 44]. CRKP has become a serious human health-threat spreading worldwide with rapid and growing rates [3]. Also, CRKP is the leading cause of many nosocomial infections and high mortality rates [5, 45]. Therefore, investigation, inhibition, or prevention of carbapenem resistance (CR) mechanisms in CRKP have become essential for research. That is what the current study tried to achieve aiming to clarify the underlying phenotypic and genotypic mechanisms of CR in *K. quasipneumoniae* ATCC 700603 (formerly *K. pneumoniae* K6) to compensate for them later on. CR is mainly mediated either by specific enzymatic production of carbapenemases, common general non-specific mechanisms of efflux increase, and/or porins deficiency relevant to multi-drug resistance [5, 46–48]. It worth mentioning that the final acquired CR can be aroused from synergism between two or more resistance mechanisms.

In the present study, we successfully evolved AMR in the *K. quasipneumoniae* ATCC 700603 experimentally via MEM-induced stress which yielded the fully MEM-resistant G20 cells from the MEM-sensitive parent G0 cells. The important signs indicating MEM adaptation and resistance acquisition were the rise of MEM-MIC values far exceeding the CLSI breakpoint along with slight retardation of cells’ growth rates playing a key role in the resistance emergence [49]. Interestingly, the acquired AMR did not limit only to MEM, but also expanded to include other classes of β-lactam antibiotics by cross-resistance or cross-protection phenomenon [2, 47]. Similar results of cross-resistance of *K. pneumoniae* isolates were obtained from the study made by Schumacher and his co-workers [50]. It is worth mentioning that the acquired AMR did not pass to different antibiotic classes such as aminoglycosides and quinolones. Thus, the AMR evolution could be mapped to a specific CR mechanism, not to the common general ones. This deduction has been confirmed by the consecutive phenotypic and genotypic experiments.

With regards to microbial growth, the final optical density of G20 cells after 18 h of cultivation was almost the same in absence or presence of MEM (0.511—0.539). Moreover, the maximum specific growth rate of G20 cells was almost the same in absence and presence of MEM stress, except for the highest MEM concentration where the *μ*_*max*_ was reduced by 21% from 0.52 to 0.41 1/h (generation time increased from 1.3 h to 2.2 h). This reduction is reasonable since the cells exert more energy in over expressing the genes encoding the defense mechanisms (β-lactamases) rather than for the growth. Concurrently, the main β-lactamase encoding gene was upregulated by 3.12-fold, resulting in a considerable ability of the adapted cells to overcome the MEM toxicity.

Carbapenemase production was detected through three phenotypic tests including nitrocefin hydrolysis, MHT, and mCIM. The fully stressed cells of G20 exhibited positive carbapenemase-production results in all previously mentioned tests assuring their successful acquisition of MEM resistance. Furthermore, these results were also supported chemically by HPLC analysis of MEM levels. The sharp decrease in the AUC of MEM peak indicated its enzymatic hydrolysis in the tested strain.

For investigation of other induced CR mechanisms, all *K. quasipneumoniae* generations were fluorescent at the lowest concentration of EtBr, 0.5 µg/ml, demonstrating their lack of efflux activity [48]. Hence, the absence of efflux activity especially with the fully stressed G20 cells further explained why their cross-resistance did not expand to different antibiotic classes of aminoglycosides and quinolones, as previously discussed.

One of the most common methods in which bacteria confront stress in their environments is to alter their morphology [51]. Hence, exposure to sub-MICs of MEM can lead to variable morphological changes that may play a role in bacterial survival from MEM-envelop stress [7]. Cell wall-related morphological adaptations occurred definitely as MEM is one of the β-lactam antibiotics affecting cell growth through inhibition of cell wall biosynthesis [52]. Similar results obtained from adaptation of *K. pneumoniae* isolates to benzalkonium chloride, a common disinfectant and preservative [48]. Also, comparable results were obtained after treating cells of *K. pneumoniae* either with the same concentration of MEM tested in our study, 8 µg/ml, [52], or with higher concentrations such as 16, and 20 µg/ml [53, 54].

Furthermore, biofilm formation can be involved in the bacterial adaptation responses to different stresses. Thus, the biofilm-forming capability of different *K. quasipneumoniae* generations was assessed. The biofilm matrix acts as an important virulence factor allowing the increase in bacterial resistance to most conventional antibiotics [55, 56]. However, the direct correlation between biofilm production and AMR evolution is fully uncertain and still debated [32]. MDROs don't need to be strong biofilm producers than non-MDROs, and vice versa [57]. Di Domenico and his co-workers proved that most ESKAPE pathogens revealing comparable levels of biofilm production were found in both MDROs and non-MDROs with no significance between the two groups [55]. Likewise, exposure of bacterial cells to antibiotic stress at sub-MICs is not relevant to biofilm formation [58]. In our study, there was a negative correlation between sub-MICs of MEM and biofilm formation in *K. quasipneumoniae*. In agreement with our results, Van Laar and his colleagues showed sub-MICs of MEM but not imipenem to inhibit biofilm formation in *K. pneumoniae* [31]. Similarly, an inverse relation between carbapenem resistance and strong biofilm formation was confirmed by Cusumano’ study in which the propensity of carbapenem-resistant *K. pneumoniae* (CRKP) to form strong biofilms was decreased by 91% [59]. Recently, a study conducted on 80 isolates of *K. pneumoniae* showed that CRKP strains had weak biofilm-forming ability relative to carbapenem-sensitive ones [60]. This inverse relation between AMR and biofilm formation can be considered as a trade-off for bacterial survival [59].

In contrary to our results, a positive correlation between MEM resistance and biofilm formation was reported [51, 57, 61]. Also, antibiotic sub-MICs trigger biofilm formation in some other organisms like *E. faecalis* [32], and *S. aureus* [62]. AMR acquisition may promote or compromise the bacterial biofilm-forming capability referring to many factors such as culture medium characteristics, growth conditions, strain type, antibiotic used and its dose (sub-MIC), method used in biofilm quantifications [57, 63].

Based on qRT-PCR data, it is obvious that the most significantly upregulated gene was *bla*_*OXA-2*_, a carbapenemase encoding gene belonged to class D β-lactamases, oxallinases [64]. The expression level of *bla*_*OXA-2*_ was estimated by a 3.12-fold increase in G20 relevant to G0 assuring carbapenemases’ overproduction [65]. It is worth mentioning that overexpression of carbapenemases by *K. quasipneumoniae* develops its resistance to nearly all β-lactam antibiotics including carbapenems. Simultaneously, the decreased influx of MEM mediated by the significant downregulation of *ompK35* estimated by 0.76-fold decrease could have a role in the total acquired CR. Further phenotypic analysis of the outer membrane proteins/porins via sodium dodecyl sulphate–polyacrylamide gel electrophoresis (SDS-PAGE) is recommended and required for validation. Conversely, the expression levels of most efflux pump genes tested were downregulated indicating a lack of efflux activity as approved phenotypically. Thus, the major CR mechanism induced inside *K. quasipneumoniae* exposed to MEM stress can be referred to the overexpression of *bla*_*OXA-2*_ genes leading to production of carbapenemases as a specific enzymatic mechanism [66]. Although *bla*_*OXA-2*_ gene is harbored in the plasmid *pKQPS2* inside the *K. quasipneumoniae* parent cells, HGT of *bla*_*OXA-2*_ genes hasn’t any role in acquisition of CR. This can be proved as follows, *K. quasipneumoniae* parent cells of G0 and cells of G20 positive control (in absence of MEM) are MEM-sensitive. Thus, the expression levels of *bla*_*OXA-2*_ genes needed to be significantly elevated for induction and acquisition of CR, what is achieved by the MEM stress along the different generations of *K. quasipneumoniae* cells.

## Conclusion

The present study mimics an environmental condition where cells are exposed to low levels of antibiotics from anthropogenic sources resulting in evolved AMR variants. Only 20 days were enough to convert *K. quasipneumoniae* from MEM-sensitive to MEM-resistant strain. Successive exposure of carbapenem-sensitive *K. quasipneumoniae* ATCC 700603 to MEM sub-MICs initiated a cascade of phenotypic and genotypic alterations relevant to the AMR evolution. It is worth mentioning that all phenotypic and genotypic changes confirmed that the acquired CR aroused mainly from MEM degradation by carbapenemases. Notably, this acquired MEM resistance expanded to other β-lactam antibiotics with cross-resistance. This study showed that AMR can be simply achieved through low-level antibiotic exposure, as naturally occurred, without any acquisition of resistance genes via the traditional HGT. Hence, rational use of antibiotics is necessary for decreasing bacterial antibiotic exposure and so suppressing the evolution and development of AMR worldwide.

## Supplementary Information

Supplementary Material 1.

Supplementary Material 2.

## Data Availability

All data generated or analysed during the study are included in this published article and its supplementary information files.

## Abbreviations

MEM: Meropenem
MIC: Minimum inhibitory concentration
Sub-MIC: Sub the minimal inhibitory concentration
AMR: Antimicrobial resistance
MDROs: Multi-drug resistant organisms
CR: Carbapenem resistance
HGT: Horizontal gene transfer
CRKP: Carbapenem-resistant *Klebsiella pneumoniae*
ARGs: Antibiotic resistant genes
CLSI: Clinical and laboratory standard institute
NB: Nutrient broth
G: Generation
CAMHB: Cation-adjusted Muller Hinton Broth
PBS: Phosphate buffered saline
TSB: Tryptone Soya Broth
TSA: Tryptone Soya Agar
LB: Luria Bertani
OD: Optical density
MHA: Muller Hinton Agar
MHT: Modified Hodge Test
mCIM: Modified carbapenem inactivation method
RP-HPLC: Reversed phase-high performance liquid chromatography
DAD: Diode array detector
CFS: Cell-free supernatants
AUC: Area under the curve
EtBr: Ethidium bromide
SEM: Scanning Electron Microscopy
CV: Crystal violet
qRT-PCR: Quantitative real time-polymerase chain reaction
Ct: Cycle threshold
