# Supplementary material for: Fundamental changes in the antimicrobial resistance profile of Klebsiella quasipneumoniae ATCC 700603 in response to meropenem stress
Source: BMC Microbiol. 2025 Jun 26;25:369. doi: 10.1186/s12866-025-04100-6 (PMC12199486; doi:10.1186/s12866-025-04100-6)
Supplement: Supplementary file 1 — Supplementary Material 1. [file 12866_2025_4100_MOESM1_ESM.pdf]

**Electronic Supplementary Information (ESI)**

# Fundamental Changes in The Antimicrobial Resistance Profile of *Klebsiella quasipneumoniae* ATCC 700603 in Response to Meropenem Stress

***Mai H. Elmahdy*, Ahmed F. Azmy, Tarek Dishisha, Ahmed O. El-Gendy, Mohamed Sebak***

*Department of Pharmaceutical Microbiology and Immunology, Faculty of Pharmacy, Beni-Suef University, 62514 Beni-Suef, Egypt.*

***Corresponding author**

E-mail: mai.elmahdy@pharm.bsu.edu.eg

Telephone: +201096656500

**Authors' details**

| E-mail | ORCID |
|---|---|
| Mai.Elmahdy@pharm.bsu.edu.eg | 0000-0002-7324-997X |
| Ahmed.Abdelaziz@pharm.bsu.edu.eg | 0000-0002-7347-9564 |
| Tarek.Dishisha@pharm.bsu.edu.eg | 0000-0001-8644-2640 |
| Ahmed.Elgendy@pharm.bsu.edu.eg | 0000-0002-0980-5185 |
| Mohamed.Sebak@pharm.bsu.edu.eg | 0000-0002-9229-2965 |

**Table S1.** Detection of MIC breakpoints of meropenem along different generations of *Klebsiella quasipneumoniae* ATCC 700603 by broth microdilution method.

| **Generation number** | **SubMIC MEM (Stress concentration) (µg/ml)** | **MIC of MEM against *Klebsiella quasipneumoniae* 700603 (µg/ml)** |
|---|---|---|
| G0 Positive control | ----------- | ≤ 0.125 |
| G0 | ----------- | ≤ 0.125 |
| G1 | 0.05 | ≤ 0.125 |
| G2 | | ≤ 0.125 |
| G3 | 0.1 | 1/4 |
| G4 | | 1 |
| G5 | 0.2 | 1 |
| G6 | | 1 |
| G7 | 0.3 | 1 |
| G8 | | 1 |
| G9 | 0.4 | 1 |
| G10 | | 4 |
| G10 Positive control | -------- | ≤ 0.125 |
| G11 | 0.5 | 4 |
| G12 | | 4 |
| G13 | 1 | 4 |
| G14 | | 8 |
| G15 | 2 | 8 |
| G16 | | 16 |
| G17 | 4 | 16 |
| G18 | | 16 |
| G19 | 8 | 32 |
| G20 | | 32 |
| G20 Positive control | -------- | ≤ 0.125 |

**Table S2.** values of $\mu_{max}$ of different generations of *Klebsiella quasipneumoniae* ATCC 700603 at different concentrations of MEM.

| **MEM Concentrations** | **Maximum specific growth rates of different *Klebsiella quasipneumoniae* generations ($\mu_{max}$ in 1/h)** | | | | |
|---|---|---|---|---|---|
| | G0 | G5 | G10 | G15 | G20 |
| **0 µg/ml** | 0.484 ±0.00 | 0.519 ±0.01 | 0.492 ±0.01 | 0.516 ±0.00 | 0.527 ±0.01 |
| **0.4 µg/ml** | 0.005 ±0.00 | 0.210 ±0.01 | 0.497 ±0.01 | 0.509 ±0.01 | 0.524 ±0.01 |
| **8 µg/ml** | 0.005 ±0.00 | 0.002 ±0.00 | 0.003 ±0.00 | 0.003 ±0.00 | 0.413 ±0.02 |

**Table S3.** Primer design for AMR genes selected after using RGI tool in CARD database

| Gene | Primer | Sequence | Product size | Tm | Ta | PCR conditions | Origin of AMR gene |
|---|---|---|---|---|---|---|---|
| ***bla*$_{OKP-B-7}$** | OKP-B-7 (F) | GTTATGTTCGCCTGTGCCTT | 217 | 55.8 | 54°C | 1.Initial denaturation at 95°C for 5 min. | Chromosome |
| | OKP-B-7 (R) | CCGCAGAGCAGCACTTTAAA | | 56 | | | |
| ***bla*$_{OXA-2}$** | OXA-2 (F) | GGTAGAACATCAGCGCTTGG | 201 | 56.1 | | 2. Denaturation at 95°C for 1 min. | Plasmid PKQPS2 |
| | OXA-2 (R) | ACGATTGCCTCCCTCTTGAA | | 56.3 | | | |
| ***bla*$_{AMP-C}$** | ampC (F) | CCGTTTATGAGCCCGAACTG | 232 | 56 | | 3. Annealing at 54°C for 30 sec. | Chromosome |
| | ampC (R) | CCGGAAGAGTAGGACCACTG | | 56.8 | | | |
| ***ompK35*** | ompK35 (F) | AGCGCAATATTCTGGCAGTG | 248 | 55.9 | | 4. Extension at 72°C for 1 min. | Chromosome |
| | ompK35 (R) | GGCGTCCATGTTGTATTCCC | | 56.2 | | | |
| ***ompK37*** | ompK37 (F) | GACCAGATGACCCAGACCAA | 158 | 56.6 | | 5. Final extension at 72°C for 10 min. | Chromosome |
| | ompK37 (R) | TTTTGCGTTTTGTTGGCCAC | | 55.4 | | | |
| ***marA*** | marA (F) | ATTCCCTCGGCCAGTACATC | 159 | 56.7 | | | Chromosome |
| | marA (R) | GGGGCGGAACATCGAAATAG | | 55.9 | | | |
| ***H-NS*** | H-NS (F) | GAGCGCCGTGAAGAAGAAAA | 177 | 55.9 | | | Chromosome |
| | H-NS (R) | GTATTTAGCCGGACGTGCAG | | 56 | | | |
| ***Lpt-D*** | LptD (F) | CACTCCGGGGTTATCGATCA | 189 | 56.3 | | | Chromosome |
| | LptD (R) | GACCTGGAACTGCTTGGTTG | | 56.3 | | | |
| ***acrR*** | acrR (F) | CCGGCAACTGATTCTGGATG | 225 | 56.2 | | | Chromosome |
| | acrR(R) | GAGAGTGGATCGTTGGGGAA | | 56.6 | | | |
| ***bla*$_{TEM-1}$** | TEM-1(F) | GTGCACGAGTGGGTTACATC | 172 | 56 | | | --------------- |
| | TEM-1(R) | GAATAGTGTATGCGGCGACC | | 55.8 | | | |
| ***bla*$_{SHV-18}$** | SHV-18(F) | GGATCTGGTGGACTACTCGC | 217 | 57.1 | | | Plasmid PKQPS2 |
| | SHV-18(R) | CGCCTCATTCAGTTCCGTTT | | 55.7 | | | |

**Table S4.** Primer design for AMR genes selected after using RGI tool in CARD database

| Genes' names | Primer Pairs | Primer sequence | Product size (bp) | Ta | PCR Conditions |
|---|---|---|---|---|---|
| ***bla*** $_{IMP}$ | IMP(F) | TCGTTTGAAGTTAACG | 568 | 46.5°C | 1.Initial denaturation at 95°C for 5 min.<br>2. Denaturation at 95°C for 1 min.<br>3. Annealing at Ta for 45 sec.<br>4. Extension at 72°C for 1 min.<br>5. Final extension at 72°C for 10 min. |
| | IMP(R) | ATGTAAGTTTCAAGAGTGATGC | | | |
| ***bla*** $_{VIM}$ | VIM(F) | CGTGTTTGGTCGCATATCGCAA | 502 | 58°C | |
| | VIM(R) | ATTCAGCCAGATCGGCATCGG | | | |
| ***bla*** $_{NDM}$ | NDM(F) | GGTTTGGCGATCTGGTTTTC | 521 | 54°C | |
| | NDM(R) | CGGAATGGCTCATCACGATC | | | |
| ***bla*** $_{OXA-48}$ | OXA-48(F) | GCTTGATCGCCCTCGATT | 238 | 54°C | |
| | OXA-48(R) | GATTTGCTCCGTGGCCGAAA | | | |
| ***bla*** $_{KPC}$ | KPC(F) | CATTCAAGGGCTTTCTTGCTGC | 498 | 54°C | |
| | KPC(R) | ACGACGGCATAGTCATTTGC | | | |

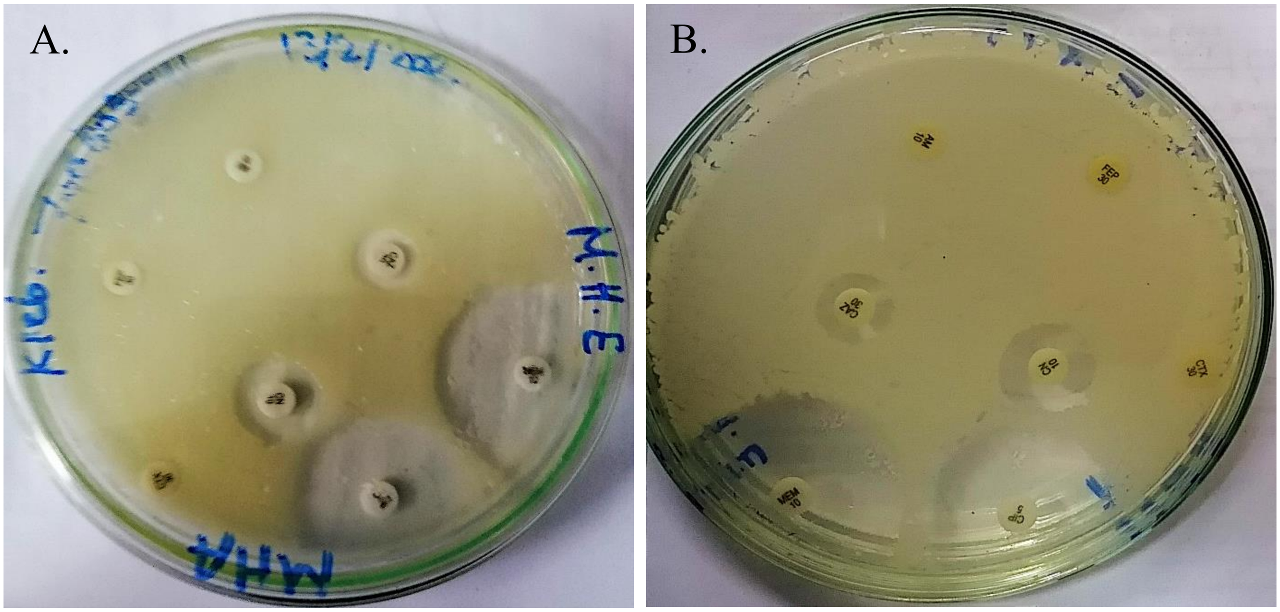


**Fig. S1.** Detection of the antibiogram of parent strain (G0) cells of *Klebsiella quasipneumoniae* ATCC 700603 using the Kirby-Bauer disk diffusion method. A. front of the plate. B. behind of the plate. The sensitivity test showed that *Klebsiella quasipneumoniae* ATCC 700603 was sensitive (S) to ciprofloxacin, meropenem, and gentamicin while being resistant (R) to ampicillin, cefotaxime, cefepime, and ceftazidime.

| **Antibiotic** | **Diameter of ZOI (mm) / *Klebsiella quasipneumoniae* ATCC 700603** |
|---|---|
| Ciprofloxacin (CIP) | 30----S |
| Gentamicin (CN) | 15----S |
| Meropenem (MEM) | 34----S |
| Ampicillin (AM) | 6----R |
| Cefotaxime (CTX) | 6----R |
| Cefepime (FEP) | 7----R |
| Ceftazidime (CAZ) | 11----R |

**Figure S2**

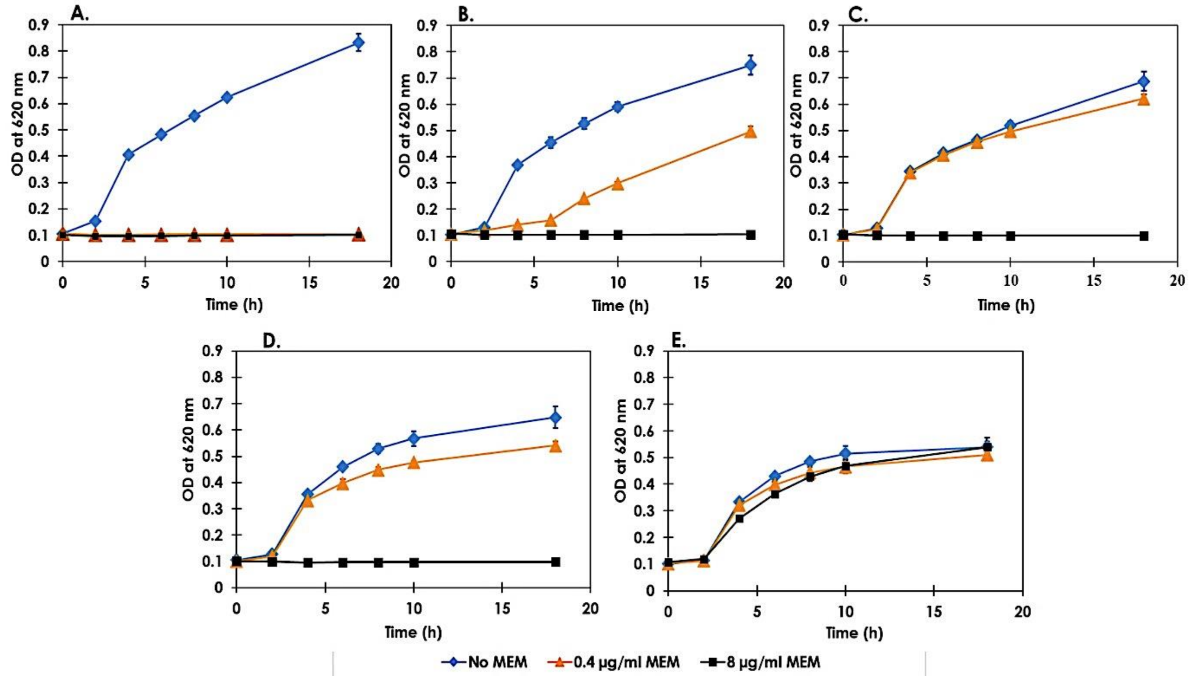


**Fig. S2.** Growth curves of different of *Klebsiella quasipneumoniae* ATCC 700603 generations (G) in absence (◆) and presence of 0.4 (▲) or 8 (■) µg/ml meropenem (MEM) in culture medium (stress conditions). A) Parent cells - G0, B) G5, C) G10, D) G15, E) G20.

**Figure S3**

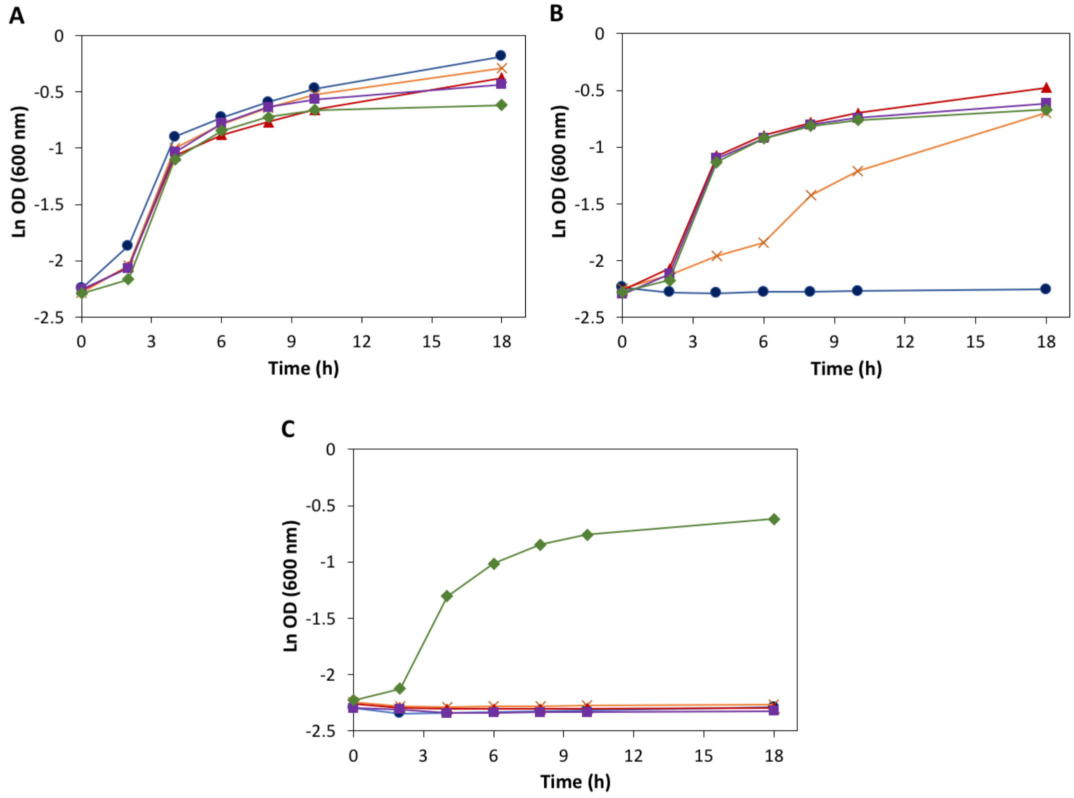


**Figure S3.** The microbial growth represented as Ln(OD) versus the cultivation time for wild-type and adapted generations (G) of *K. quasipneumoniae* ATCC 700603, in presence of (A) 0 µg/ml of MEM, (B) 0.4 µg/ml of MEM, and (C) 8 µg/ml of MEM; (●) for G0, (×) for G5, (▲) for G10, (■) for G15, and (◆) for G20.
